# Management of delayed colonic perforation after endoscopic submucosal dissection: the over-the-scope clip way

**DOI:** 10.1016/j.vgie.2024.12.005

**Published:** 2024-12-26

**Authors:** Yohei Minato, Krishanu Banik, Toshifumi Iida, Nao Takeuchi, Yoshiaki Kimoto, Yuki Kano, Kohei Ono, Ken Ohata

**Affiliations:** 1 Department of Gastrointestinal Endoscopy, NTT Medical Center Tokyo, Tokyo, Japan; 2 Department of Medicine, College of Medicine and JNM Hospital, Kalyani, West Bengal, India

Delayed perforation is a rare and serious adverse event after endoscopic submucosal dissection (ESD), developing within 48 hours after the procedure without any signs of perforation or muscle injury during ESD.^1^ Delayed perforation after colonic ESD is often more serious than gastric ESD because of high chance of peritonitis as the result of seepage of digestive enzymes and bacteria-rich colonic fluid.^2^ Endoscopy for delayed perforations can be dangerous because of the risk of barotrauma and further aggravation of the perforation.^3^ Because of the risk of rapidly developing peritonitis and limited definitive nonsurgical options, it often is treated by emergency surgery.^4^ We report a case of delayed colonic perforation after ESD being successfully managed by endoscopic placement of an over-the-scope clip (OTSC).

## Case report

A 71-year-old male patient was diagnosed with a 40-mm superficial neoplastic lesion in the proximal ascending colon (Paris 0-IIa). We removed it en bloc by ESD. The procedure time was 35 minutes. We used a high-frequency generator (VIO 300 D; Erbe, Tübingen, Germany) as the current source; a mixture of normal saline, 1:1000 epinephrine, indigo carmine, and 0.4% sodium hyaluronate (Ksmart; Olympus, Tokyo, Japan) as the lifting solution; a disposable endoscopic injector needle (Medico's Hirata, Inc, Osaka, Japan) as the injector needle; a Dual-knife (Olympus) for mucosal incision and submucosal dissection; and EZ clips (short clip, HX-610-135S; Olympus) and hemostatic forceps (Raicho2; Kaneka Corp, Osaka, Japan) for hemostasis.

**Figure 1:**
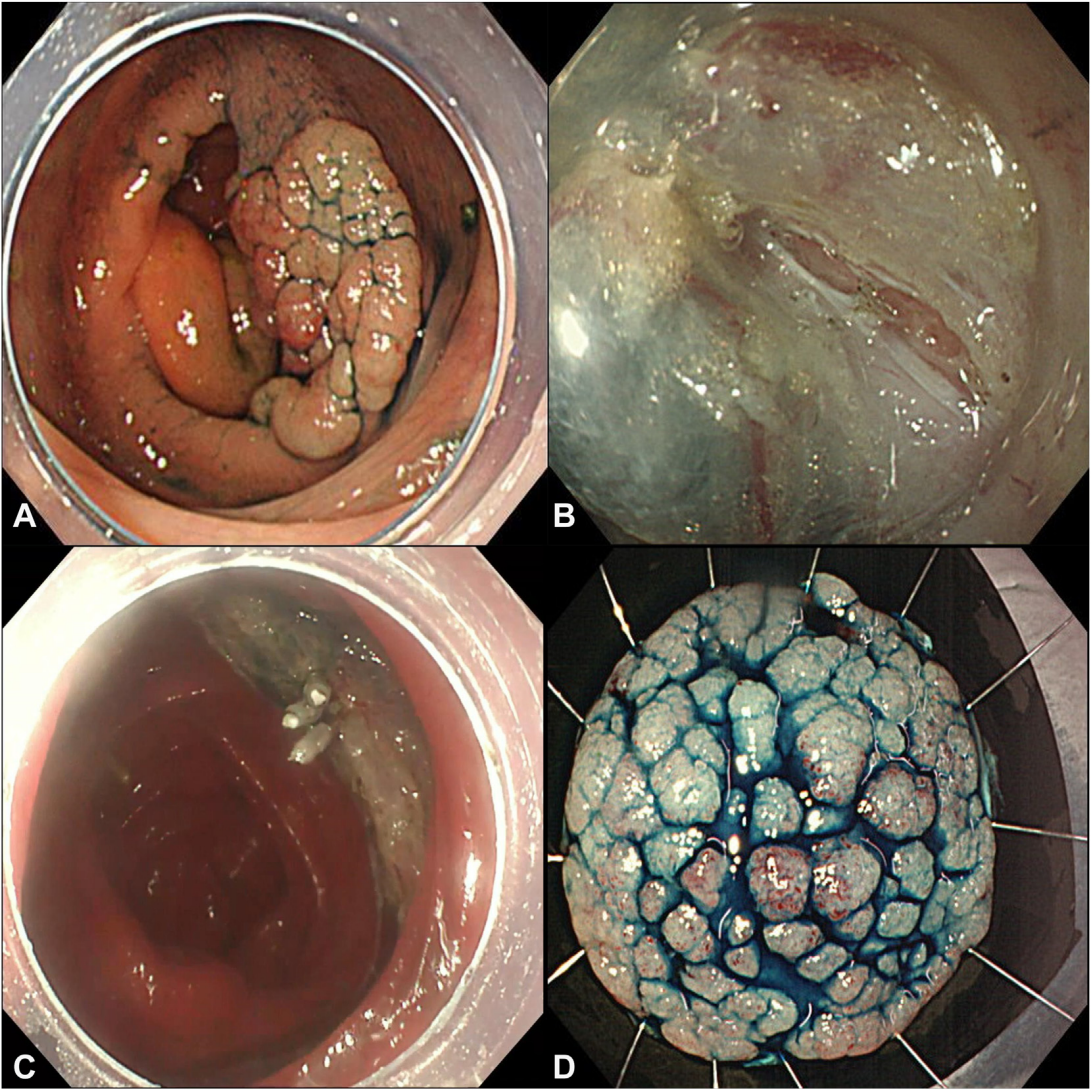
ESD for a lesion in the ascending colon. **A,** A 40-mm superficial neoplastic lesion in the proximal ascending colon. **B,** Partial muscular layer damage was observed in the ulcer base. **C,** The area with muscular layer damage was closed with clips. **D,** En bloc specimen (34 mm × 33 mm). *ESD*, Endoscopic submucosal dissection.

The resected specimen was 34 × 33 mm in size (high-grade tubular adenoma pVM0, pHM0 on histopathologic examination). After resection, we noticed oozing blood from the center of the defect and muscle injury. We used 3 EZ clips for hemostasis and reinforcement of the muscle layer injury (Fig. 1). The next morning, he developed sudden-onset right lower abdominal pain. It was initially mild and became severe in the evening. There was an increased inflammatory response (raised neutrophils and C-reactive protein). The abdomen was soft and had no rebound tenderness, so we determined that generalized peritonitis had not developed.

## Procedure and outcome

**Figure 2:**
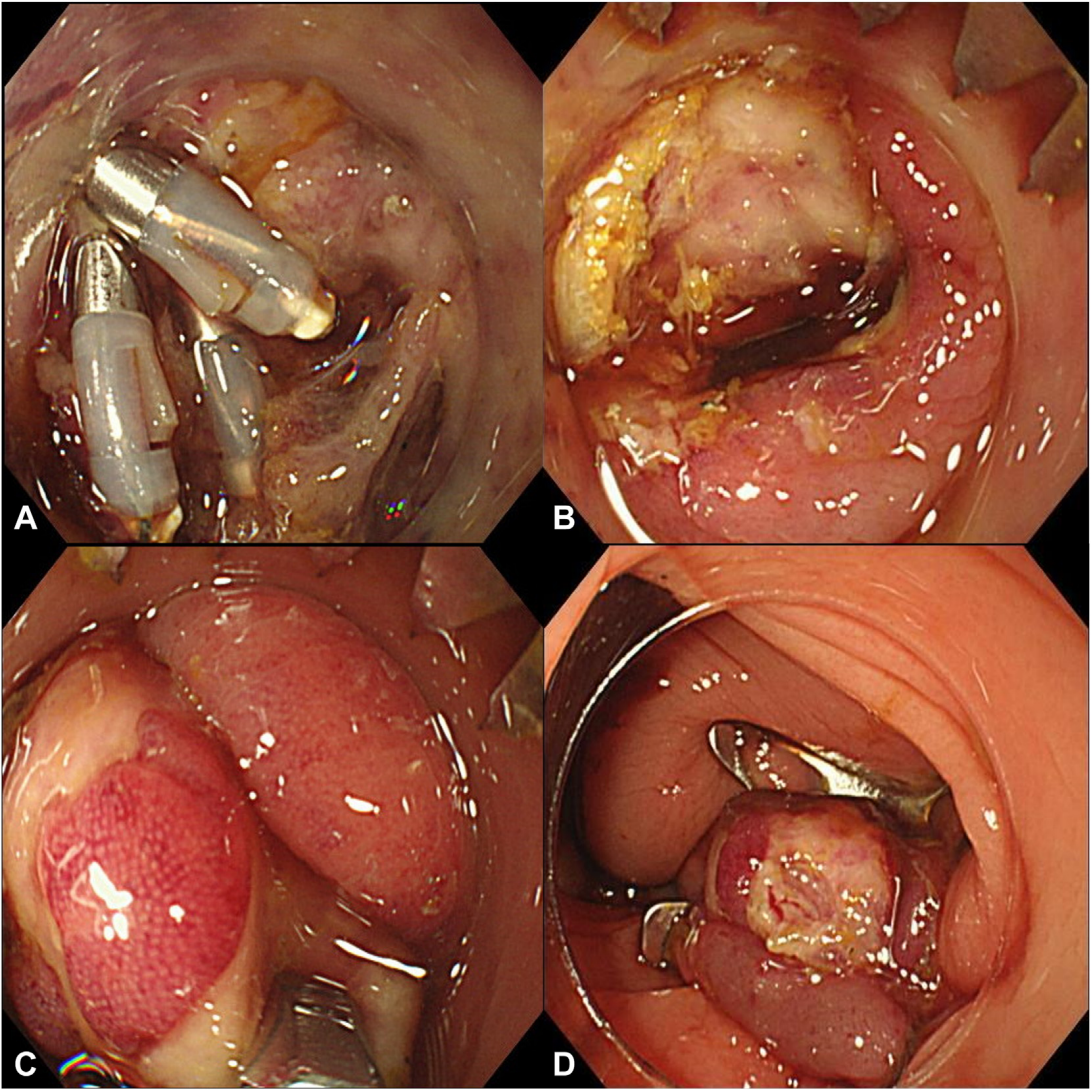
Post-ESD delayed colonic perforation closed by an OTSC. **A,** A perforation site was observed in the post-ESD ulcer, distinct from the location where clips were applied during the ESD procedure. **B,** The clip was removed to facilitate the placement of the OTSC, and the OTSC was set at the perforation site. **C,** The surrounding tissue of the perforation was grasped with forceps and pulled into the distal attachment. **D,** The perforation site and the surrounding ulcer base were closed with an OTSC. *ESD*, Endoscopic submucosal dissection, *OTSC*, over-the-scope clip.

We suspected delayed perforation. Although colonoscopy in cases of perforation is risky and generally contraindicated due to the potential for enlarging the perforation,^5^ we proceeded because the patient became symptomatic within 24 hours and was continuously kept fasting, and no signs of peritonitis developed. After fully explaining the procedure to the patient, emergency colonoscopy revealed a perforation approximately 6 mm in size (Fig. 2). The tissue at the ulcer base was friable and covered with a thick white exudate, and the surrounding area was edematous, so we judged that closure with conventional clips would be difficult. We determined that using the OTSC for secure closure was a beneficial approach, even if it required withdrawing and reinserting the scope. A 10-mm t-type OTSC (Ovesco, Tübingen, Germany) with small spikes and blunted edges, designed for both compression and tissue anchoring, was selected. We removed previously applied clips, as they might have hindered successful OTSC closure. The perforation base was then grasped and pulled inside using conventional grasping forceps along with suction to ensure a good margin, leaving no gaps, and the OTSC was applied. This successfully closed the perforation (Video 1, available online at www.videogie.org). The procedure time for closure of delayed perforation from initial insertion was 30 minutes. After the procedure, the patient was managed via fasting and intravenous infusion of antibiotics from day 2 to 6. The patient resumed eating on day 7 and was discharged on day 8.

## Discussion

Given the rarity of post-ESD delayed colonic perforation, the tendency for rapid development of peritonitis, and the lack of an established treatment algorithm, management should be individualized. Our experience suggests that emergency endoscopy is valuable for diagnosing delayed perforation when panperitonitis is absent. As in our case, conventional clip closure may not always be feasible because of tissue friability, necrosis, and thickening at the ulcer base around the perforation site. An OTSC can be an effective option, bridging the gap between clip closure and surgery, as it can successfully grasp adequate tissue around the perforation site despite tissue fragility, thickening, and necrosis.

## Conclusions

If the patient is in the early postoperative stage without signs of peritonitis, closing the perforation site with the OTSC can be an option to avoid emergency surgery.

## Patient Consent

The patient in this article has given written informed consent to publication of the case details.

## Disclosure

All authors disclosed no financial relationships.

## Supplementary data

Video 1 OTSC closure of post-ESD delayed colonic perforation.

## References

[1] Zhou G.-Y.-J., Hu J.-L., Wang S. (2020). Delayed perforation after endoscopic resection of a colonic laterally spreading tumor: a case report and literature review. World J Clin Cases.

[2] Taku K., Sano Y., Fu K.-I. (2007). Iatrogenic perforation associated with therapeutic colonoscopy: a multicenter study in Japan. J Gastroenterol Hepatol.

[3] Dellon E.S., Hawk J.S., Grimm I.S. (2009). The use of carbon dioxide for insufflation during GI endoscopy: a systematic review. Gastrointest Endosc.

[4] Fujihara S., Mori H., Kobara H. (2013). The efficacy and safety of prophylactic closure for a large mucosal defect after colorectal endoscopic submucosal dissection. Oncol Rep.

[5] Kawashima K., Hikichi T., Fujiwara T. (2018). Delayed perforation after endoscopic submucosal dissection for mucosal colon cancer: a conservatively treated case. Fukushima J Med Sci.

